# Expression of Heterologous *OsDHAR* Gene Improves Glutathione (GSH)-Dependent Antioxidant System and Maintenance of Cellular Redox Status in *Synechococcus elongatus* PCC 7942

**DOI:** 10.3389/fpls.2020.00231

**Published:** 2020-03-03

**Authors:** Young-Saeng Kim, Seong-Im Park, Jin-Ju Kim, Joseph S. Boyd, Joris Beld, Arnaud Taton, Kyoung-In Lee, Il-Sup Kim, James W. Golden, Ho-Sung Yoon

**Affiliations:** ^1^Research Institute for Dok-do and Ulleung-do, Kyungpook National University, Daegu, South Korea; ^2^School of Life Sciences, BK21 Plus KNU Creative BioResearch Group, Kyungpook National University, Daegu, South Korea; ^3^Department of Biology, Kyungpook National University, Daegu, South Korea; ^4^Division of Biological Sciences, University of California, San Diego, La Jolla, CA, United States; ^5^Department of Microbiology and Immunology, College of Medicine, Drexel University, Philadelphia, PA, United States; ^6^Biotechnology Industrialization Center, Dongshin University, Naju, South Korea; ^7^Advanced Bio Resource Research Center, Kyungpook National University, Daegu, South Korea

**Keywords:** antioxidants, antioxidant-related enzymes, cyanobacterium, dehydroascorbate reductase, oxidative stress

## Abstract

An excess of reactive oxygen species (ROS) can cause severe oxidative damage to cellular components in photosynthetic cells. Antioxidant systems, such as the glutathione (GSH) pools, regulate redox status in cells to guard against such damage. Dehydroascorbate reductase (DHAR, EC 1.8.5.1) catalyzes the glutathione-dependent reduction of oxidized ascorbate (dehydroascorbate) and contains a redox active site and glutathione binding-site. The *DHAR* gene is important in biological and abiotic stress responses involving reduction of the oxidative damage caused by ROS. In this study, transgenic *Synechococcus elongatus* PCC 7942 (TA) was constructed by cloning the *Oryza sativa* L. *japonica DHAR* (*OsDHAR*) gene controlled by an isopropyl β-D-1-thiogalactopyranoside (IPTG)-inducible promoter (*Ptrc*) into the cyanobacterium to study the functional activities of *OsDHAR* under oxidative stress caused by hydrogen peroxide exposure. *OsDHAR* expression increased the growth of *S. elongatus* PCC 7942 under oxidative stress by reducing the levels of hydroperoxides and malondialdehyde (MDA) and mitigating the loss of chlorophyll. DHAR and glutathione *S*-transferase activity were higher than in the wild-type *S. elongatus* PCC 7942 (WT). Additionally, overexpression of *OsDHAR* in *S. elongatus* PCC 7942 greatly increased the glutathione (GSH)/glutathione disulfide (GSSG) ratio in the presence or absence of hydrogen peroxide. These results strongly suggest that *DHAR* attenuates deleterious oxidative effects via the glutathione (GSH)-dependent antioxidant system in cyanobacterial cells. The expression of heterologous *OsDHAR* in *S. elongatus* PCC 7942 protected cells from oxidative damage through a GSH-dependent antioxidant system via GSH-dependent reactions at the redox active site and GSH binding site residues during oxidative stress.

## Introduction

The maintenance of cellular redox balance requires a steady-state equilibrium between oxidants and antioxidants. Under oxidative stress, loss of detoxification and accumulation of excess reactive oxygen species (ROS) leads to cell death through disruptions to redox balance, subsequent signaling, and cell rescue systems (Boyer, 1982; Owens, 2001; Rouhier et al., 2008). In aerobic cells, ROS degradation occurs through the GSH pools, which is catalyzed by a series of antioxidative enzymes localized primarily in intracellular and extracellular areas with high ROS production (Potters et al., 2002). Dehydroascorbate reductases (DHARs) are important to the GSH-dependent reduction of dehydroascorbate (DHA) to ascorbate, and their activities are shown to affect redox homeostasis in higher plants (Shimaoka et al., 2000; Urano et al., 2000; Dixon et al., 2002; Dixon and Edwards, 2010). The presence of a *DHAR* gene has yet to be conclusively established in the cyanobacterium *Synechococcus elongatus* PCC 7942.

In previous studies, we initially predicted that the GSH-bound *Oryza sativa* L. *japonica* DHAR (OsDHAR) structure might contain a GSH binding site (Do et al., 2016). A subsequent study reported the pronounced similarity of GSH binding sites in the conserved residues of glutathione *S*-transferases (GSTs), such as thioredoxin-like sites found in different species (Abdul Kayum et al., 2018). These residues indicate that the DHARs are GST-like proteins with GSH-dependent thiol transferase activity, with a cysteine at the active site instead of serine or tyrosine (Dixon et al., 2002). In DHAR activity, the first cysteine residue in the GSH binding site reacts with the sulfur atom in the GSH mixed disulfide bridges and promotes thiol transfer (Dixon et al., 2002; Dixon and Edwards, 2010). Mutation of the first cysteine residue induces significant losses of catalytic efficiency and GSH binding affinity at GSH binding site residues during abiotic stresses (Tang and Yang, 2013; Do et al., 2016).

There is also growing evidence that overexpression of *DHAR* enhances tolerance to many abiotic stresses. Overexpression of wheat *DHAR* conferred protection from ozone in transgenic tobacco (Chen and Gallie, 2005), and reinforcement of tobacco with the human *DHAR* gene increased tolerance to low temperatures and salt stress (Kwon et al., 2003). Overexpression of rice *DHAR* increased salt tolerance in *Arabidopsis* (Ushimaru et al., 2006) and resistance to oxidative stress in *Escherichia coli* (Shin et al., 2008). Overexpression of *Arabidopsis* cytosolic *DHAR* enhanced tolerance to drought and ozone stress in tobacco plants (Eltayeb et al., 2006), and overexpression of homologous *OsDHAR* containing a redox active site and GSH binding site residues in transgenic rice improved environmental adaptation and rice productivity in paddy fields (Kim et al., 2013). Although the relationship between *DHAR* expression and stress tolerance in plants is supported by a great deal of evidence, there is little information concerning the effect of *DHAR* overexpression in cyanobacteria. Cyanobacteria are believed to be the ancestors of photosynthetic eukaryotes as a result of an ancient endosymbiosis (Blank, 2013). The unicellular *S. elongatus* PCC 7942 has been used extensively as a cyanobacterial model for biochemical, physiological, and genetic studies of important cellular processes, such as prokaryotic nitrate reductases, cell division, responses to nutrient depletion, iron deprivation, and environmental abiotic stresses, such as ambient temperature and light intensity (Koksharova et al., 2006).

This study was undertaken to characterize biochemical and physiological features of *OsDHAR*-mediated stress tolerance in transgenic *S. elongatus* PCC 7942 under ROS-induced oxidative stress conditions. We found that the expression of heterologous *OsDHAR* in *S. elongatus* PCC 7942 conferred resistance to oxidative stress by helping to maintain cellular redox homeostasis through a GSH-dependent antioxidant system via GSH-dependent reactions. The multifaceted approach we employed provides new insights into the mechanisms of gene family expansion and functional evolution.

## Materials and Methods

### Amino Acid Sequence Alignment

BLAST software^1^ was used to align OsDHAR with known DHAR sequences using the National Center for Biotechnology Information (NCBI) database. Amino acid sequences were as follows: *O. sativa* DHAR (OsDHAR; accession no. AAL71856.1), *S. elongatus* PCC 7942 GST (SeGST; accession no. WP_011243077.1), *Synechocystis* sp. PCC6803 GST (StGST; accession no. WP_010872521.1), *Gloeobacter violaceus* PCC 7421 GST (GvGST; accession no. WP_011143013.1), *Prochlorococcus marinus* MIT9313 GST (PcGST; accession no. WP_011124721.1), *Acaryochloris marina* MBIC11017 GST (AmGST; accession no. WP_012161393.1), and *Cyanothece* sp. ATCC 51142 GST (CtGST; accession no. WP_009546792.1). The GSTs from these cells are soluble monomeric enzymes that contain a GSH-binding redox active site and similarly functional conserved residues comprised of different amino acids in other active sites.

### Construction of Recombinant Plasmid *OsDHAR*

The *DHAR* gene from *O. sativa* L. *japonica* (accession no. AY074786.1; *OsDHAR*) coding region was amplified from cDNA using PCR with ExTaq polymerase (Takara Bio Inc., Shiga, Japan). PCR reaction conditions were as follows: initial denaturation at 95°C for 3 min, followed by 30 cycles of 95°C for 30 s, 53°C for 30 s, and 72°C for 1 min, and a final extension for 5 min at 72°C. The *OsDHAR* gene was cloned by PCR using OsDHAR-F-*Swa*I and OsDHAR-R-*Swa*I as the sense and antisense primers, respectively (Supplementary Table S2). The insertion of heterologous *OsDHAR* in *S. elongatus* PCC 7942 was constructed using the pAM4957 (pCV0069) a conjugative plasmid vector for chromosomal integration into recombinant complementation constructs at neutral site II (NS2).

Competent *E. coli* DH5α cells that had been conjugated by the *pAM4957:OsDHAR* plasmid were selected using nourseothricin (cloNAT, 50 μg mL^–1^). Clones were confirmed by sequencing, then used to conjugate *S. elongatus* PCC 7942. The plasmids harboring the *OsDHAR* encoding gene were sequenced using neutral site II (NS2) primers (Supplementary Tables S1, S2) complementary to the NS2 region to confirm proper gene ligation and direction. Finally, the resulting plasmid was assembled using the GeneArt Seamless Cloning and Assembly Kit (Life Technologies, Carlsbad, CA, United States). Plasmids harboring the *OsDHAR* encoding gene were constructed by recombinant complementation, and chromosomal integration into *S. elongatus* PCC 7942 NS2 using published protocols (Golden et al., 1987). Integration of the *pAM4957:OsDHAR* transgene into genomic DNA of the *S. elongatus* PCC 7942 was verified by PCR using Ptrc-F and OsDHAR-R primers (Supplementary Table S2) and the PCR PreMix kit (Bioneer, Daejeon, South Korea) according to the manufacturer’s instructions.

### Stress Conditions

Cyanobacteria were cultured in BG11 medium at 30°C in continuous light (80–120 μE m^–2^s^–1^) on a rotary shaker (120 rpm) for 7 days in Erlenmeyer flask. Then, the TA, EV, and WT *S. elongatus* PCC 7942 were cultured in liquid BG11 medium containing 2 mM isopropyl β-D-1-thiogalactopyranoside (IPTG) at 5 days before 2.5 mM H_2_O_2_ treatment at 7 days with shaking. The TA, and EV *S. elongatus* PCC 7942 were maintained in BG11 liquid or solid media supplemented with the appropriate antibiotics.

Initial absorbances at 730 nm (*A*_730_) were adjusted to 0.03. Aliquots of each sample were then measured to adjust them to 0.03 in BG11 medium after culturing for 18 days at 30°C with shaking in liquid microbial lab flasks (Supplementary Figure S2A). Responses to oxidative stress were evaluated by allowing cells to reach the exponential phase (*A*_730_ = 0.3–0.35) before exposing them to 2.5 mM H_2_O_2_ at 7 days with shaking, after which *A*_730_ values were monitored each day through the end of the experiment.

For the cell-spotting assay, the growth following the presence (10 days) or absence (7 days) of 2.5 mM H_2_O_2_ with 2 mM IPTG for 3 days were measured at the TA, EV, and WT *S. elongatus* PCC 7942 (*A*_730_ = 0.25) and were then serially diluted 10-fold with distilled water. The diluted samples were spotted on agar-solidified BG11 medium and incubated for 3 days at 30°C. They were then photographed or counted. All experiments were performed three or more times.

### Semi-Quantitative RT-PCR

The cDNA was reverse transcribed from total RNA using the SuperScript III kit (Life Technologies). For semi-qRT-PCR, the OsDHAR-qRT-PCR-F and OsDHAR-qRT-PCR-R (Supplementary Table S2) primer sets were used to amplify *OsDHAR*. The reaction sequence consisted of one cycle at 95°C for 3 min followed by 26 cycles at 94°C for 30 s, 54°C for 30 s, and 72°C for 40 s, and then a final extension at 72°C for 5 min. The semi-qRT-PCR amplicons were resolved on a 1.0% agarose gel by electrophoresis in 0.5X Tris/borate/EDTA buffer. The *rpoA* gene primer sets were used as a housekeeping control and were also amplified (Supplementary Table S2).

### Western Blot Analysis

Western blot analysis of the crude extracts was performed by obtaining the total protein from cyanobacteria (*A*_730_ = 0.3) with suspension in five volumes of cold extraction buffer containing 150 mM NaCl, 50 mM Tris-HCl (pH 7.3), 1 mM EDTA, 2% β-mercaptoethanol, 1 mM dithiothreitol, 1 mM phenylmethylsulfonyl fluoride (PMSF), and an equal of volume of phenol saturated with Tris-HCl (pH 7.3). Protein was then extracted from the cells as previously described (Kim et al., 2018).

Briefly, crude protein extracts were prepared using glass beads. Cells grown for 7 days were exposed to 2.5 mM H_2_O_2_ for 24 h to induce cellular oxidative stress, followed by vigorous vortexing 10 times for 1 min each at 3-min intervals on ice. The protein extracts were cleared by centrifugation at 12,000 rpm for 20 min at 4°C. Finally, protein concentrations were determined using a Pierce bicinchoninic acid protein assay kit (Thermo Scientific, Waltham, MA, United States) (Komatsu et al., 2009; Peng et al., 2011).

Protein extracts (20 μg) were separated on a 10% SDS-PAGE gel at 100 V and transferred to polyvinylidene fluoride membranes (Bio-Rad, Hercules, CA, United States). Membranes were then incubated in blocking buffer consisting of 5% non-fat skim milk and 0.02% sodium azide in Tris-buffered saline plus Tween [TBST, 10% Tween-20, 20 mM Tris-HCl (pH 7.6), and 150 mM NaCl] for 1.5 h at room temperature. Antibodies to *O. sativa* DHAR (OsDHAR) were produced in rabbits hyperimmunized using proteins purified from *E. coli* (Younginfrontier, Seoul, South Korea). The blots were washed three times for 30 min with TBST, after which they were incubated with conjugated anti-rabbit secondary antibody (Santa Cruz Biotechnology, Santa Cruz, CA, United States) diluted with blocking buffer (without 0.02% sodium azide) for 4 h at room temperature. After washing with TBST, binding of antibody to protein immobilized on the blots was visualized using the SuperSignal West Femto substrate kit (Pierce, Rockford, IL, United States) and imaged using a MultiImage II Light Cabinet (DE-500; Alpha Innotech Corporation, San Leandro, CA, United States).

### Chlorophyll Content and Dry Weight Measurement

Chlorophyll content assays were conducted by harvesting the TA, EV, and WT *S. elongatus* PCC 7942 from a 100 mL culture at an *A*_730_ of 0.3 after a 2-day exposure to 2.5 mM H_2_O_2_ in liquid BG11 medium containing 2 mM IPTG. Chlorophyll content was analyzed by treating cultures (100 mL, *A*_730_ = 0.3) with 2.5 mM H_2_O_2_ for 2 days. The cultivated cells were harvested, and the cell pellets were suspended in 90% methanol. Chlorophyll was extracted as previously described (Kim et al., 2017, 2018) with some modifications. The chlorophyll content of the cells was measured by scanning them over 260 to 800 nm using an Infinite M200 Pro microplate reader (Tecan, Männedorf, Switzerland). All assays were performed in biologically least three independent experiments. The dry weights of cells at 9 days after 2-day exposure to H_2_O_2_ were determined gravimetrically as previously described (Gorain et al., 2013; Kim et al., 2017). Other dry weight samples were measured at 14 days after growth in the presence or absence of 2.5 mM H_2_O_2_ for 7 days (Supplementary Figure S2B).

### Redox State and Measurement of Thiobarbituric Acid Reactive Substances (TBARS) Value

The intracellular levels of H_2_O_2_ in cells exposed to H_2_O_2_ with IPTG (H_2_O_2_-IPTG) for 24 h were determined using the ferrous oxidation-xylenol orange (FOX) reagent (100 μM xylenol orange, 250 μM ammonium ferrous sulfate, 100 mM sorbitol, and 25 mM sulfuric acid) by ferrous ion oxidation in the presence of the ferric ion indicator xylenol orange (Kim et al., 2011, 2018).

Cellular ROS levels were measured *in vivo* by harvesting of the TA, EV, and WT *S. elongatus* PCC 7942 from 100 mL culture (*A*_730_ = 0.3) after exposure to 2.5 mM H_2_O_2_ -IPTG with 2 mM IPTG for 2 h; these were incubated for 20 min at 30°C with 10 μM DCFHDA (Invitrogen, Carlsbad, CA, United States) in the dark (Wang and Joseph, 1999; Kim et al., 2018). Cells were then collected and examined using a model LSM700 fluorospectrophotometer (Carl Zeiss, Jena, Germany) at an excitation wavelength of 488 nm and an emission wavelength of 535 nm. Images were also captured by laser scanning confocal microscopy at an excitation wavelength of 488 nm (Huang et al., 2012). Levels of lipid peroxidation were evaluated in cells exposed to H_2_O_2_-IPTG with 2 mM IPTG for 24 h. The extent of oxidative damage to the lipids was determined by measuring malondialdehyde (MDA) content; this was achieved adding 10% trichloroacetic acid containing 0.65% 2-thiobarbituric acid (TBA) and heating at 95°C for 25 min. Finally, the MDA concentration of the resulting supernatant was measured at 532 nm (based on 600 nm) and estimated using an absorbance coefficient of 1.56 × 105, as described by Jiang and Zhang (2001) and Kim et al. (2018).

### Analyses of Antioxidants and Antioxidant-Related Enzymes Under ROS-Induced Oxidative Stress

We evaluated potential mechanisms by which the TA tolerates oxidative stress by measuring GSH/GSSG ratio as they related to total GSH content during a 24-h exposure to H_2_O_2_. The TA, EV, and WT *S. elongatus* PCC 7942 were harvested by centrifugation and then washed twice with cold phosphate-buffered saline (PBS). We then measured GST activity by adding the crude protein of samples exposed to the H_2_O_2_ stress for 24 h to a lysis buffer containing 50 mM sodium phosphate (pH 7.5), 3 mM MgCl_2_, 1 mM EDTA, 1 mM PMSF, and protease inhibitor cocktails. Total GSH content was measured as previously described (Jiang and Zhang, 2001). GST enzyme activity was detected as previously described using 1-chloro-2,4- dinitrobenzene as substrate (Dixit et al., 2015).

### qRT-PCR

An RNA extraction of 3 different samples exposed to 2.5 mM H_2_O_2_ for 24 h for biologically least three independent experiments. Samples were frozen with liquid nitrogen and ground using a mortar and pestle. Total RNA was extracted using TRIzol reagent (Takara, Japan) according to the manufacturer’s instructions, and reverse transcription reactions were performed with 2 μg total RNA using the SuperScript III First-Strand Synthesis System. The qRT-PCR analysis was performed using the TB Green Premix Ex Taq II (Tli RNaseH Plus) Kit (Takara, Japan) and QuantStudio3 Real-Time PCR system (Applied Biosystems, Foster City, CA, United States). The 20 μL reactions contained 12.5 μL of TB Green Premix Ex Taq II (Tli RNaseH Plus), 0.4 μL of ROX Reference Dye II, 200 ng of cDNA template, 0.4 μL of each primer (10 μM), and 6.26 μL of PCR-grade water. All samples were analyzed in biological triplicates as follows: initial preheating at 95°C for 30 s, followed by 40 cycles of 95°C for 3 s and 55°C for 30 s. The *rpoA* gene primer sets were used as a positive control. Fold changes in mRNA were calculated relative to the calibrator. Expression levels of GSH-related genes in Figure 6 is presented relative to that in TA strains (which expression level was considered to be 1 of WT strains; described as relative values.) under stress condition (Supplementary Figure S3). Normalization and relative quantification were conducted using the 2^–ΔΔCt^ method (Livak and Schmittgen, 2001). The primers used for qRT-PCR analysis are listed in the Supplementary Table S2.

### Statistical Analyses

All biochemical results from the TA were calculated relative to those of the EV and WT under normal conditions, which were defined as 100%. Significant differences among results for the TA, EV, and WT were determined by ANOVA. Comparisons between individual data points were performed using Student’s *t*-test, and a *P*-value of less than 0.05 was considered statistically significant. All experiments were carried out three or more times, and all results are expressed as mean ± SD.

## Results

### Sequence Analysis of OsDHAR Protein Motifs

**FIGURE 1:**
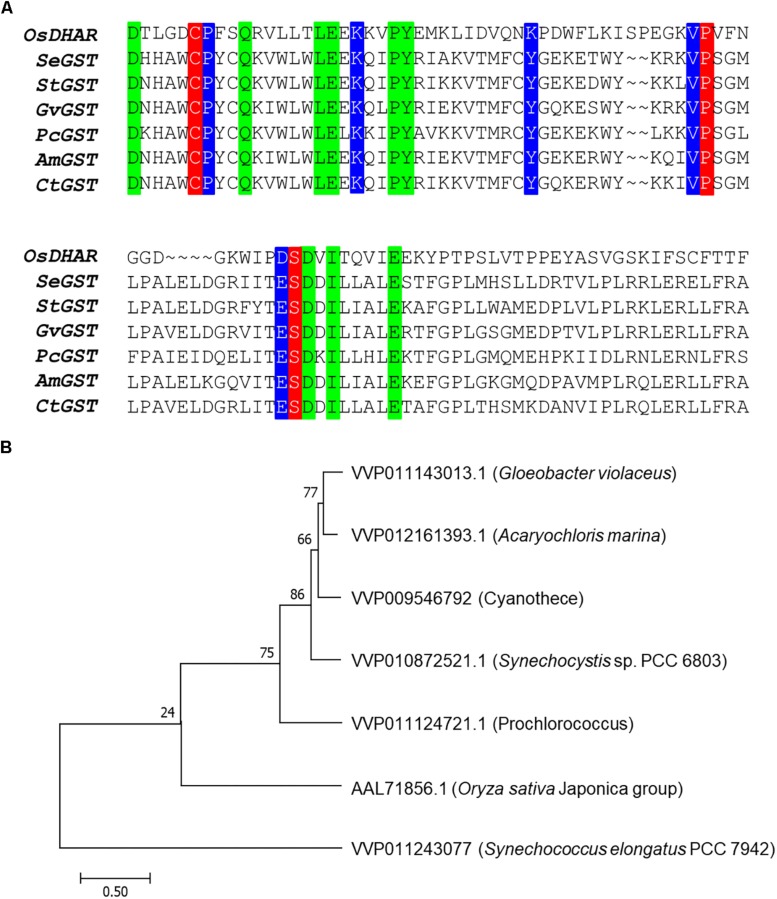
Amino acid alignment of OsDHAR and six glutathione *S*-transferase (GSTs). **(A)** Alignment showing GSH binding site and GSTs with similarly functional conserved residues. Colored boxes indicate identical residues across sequences. All contain a conserved site with GSH binding residues (red background), GSTs having similar functional conserved residues (blue background), and strictly conserved residues (green boxes). **(B)** The phylogenetic trees show the evolutionary relationship between rice DHAR and the amino acids of cyanobacterium GSTs. OsDHAR, *Oryza sativa* DHAR; SeGST, *Synechococcus elongatus* PCC 7942 GST; StGST, *Synechocystis* sp. PCC6803 GST; GvGST, *Gloeobacter violaceus* PCC 7421 GST; PcGST, *Prochlorococcus marinus* MIT9313 GST; AmGST, *Acaryochloris marina* MBIC11017 GST; CtGST, *Cyanothece* sp. ATCC 51142 GST.

The amino acid sequence motifs of OsDHAR were compared with the sequences of known GSTs to clarify the molecular properties of OsDHAR. Pairwise alignment of the sequences of OsDHAR and the other GSTs were conducted using BLAST software. OsDHAR displayed 24, 26, 25, 26, 20, and 23% similarity to SeGST, StGST, GvGST, PcGST, AmGST, and CtGST, respectively (Figure 1A, see section “Materials and Methods” for details of the GSTs). All contained a conserved site that bound GSH residues (Cys20, Pro61, and Ser74) (Figure 1A, red background) and similar functionally conserved residues (Pro21, Lys34, Lys47, Val60, and Asp73) (Figure 1A, blue background) that are likely involved in redox active site function. Other strictly conserved residues were not clearly evident (Figure 1A, green boxes). We used these results to construct a phylogenetic tree containing the *S. elongatus* PCC 7942 GST (SeGST; accession no. WP_011243077) that was 24% homologous to OsDHAR (accession no. AAL71856.1) (Figure 1B).

### Cellular Response of the *OsDHAR*-Expressing in *S. elongatus* PCC 7942 Under H_2_O_2_ Stress Conditions

**FIGURE 2:**
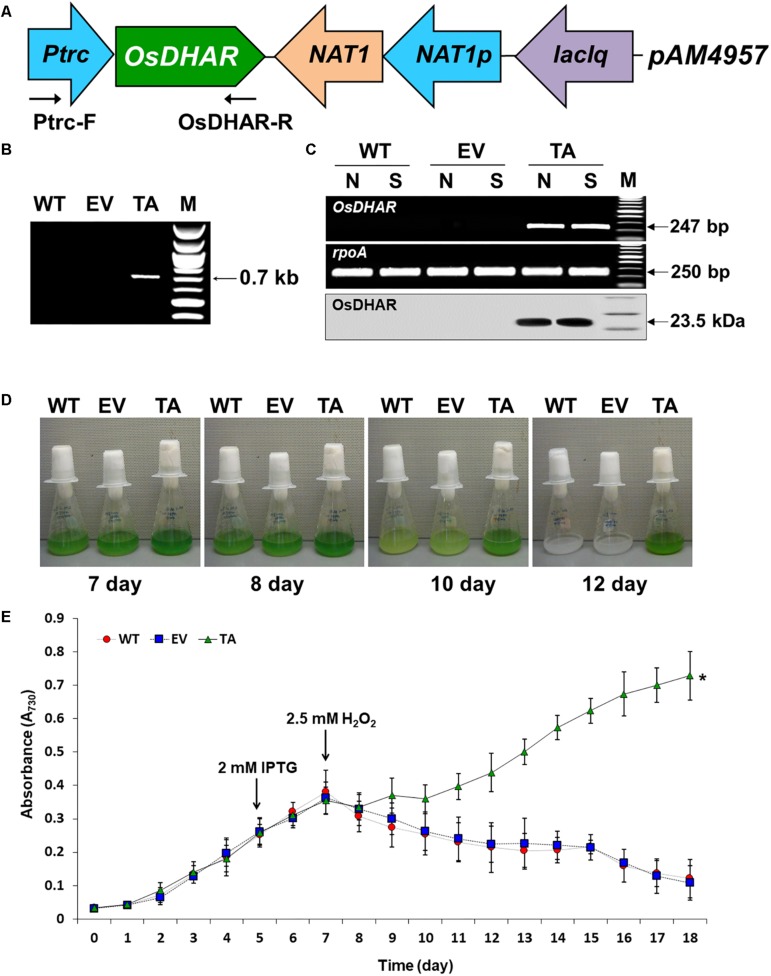
Expression of the *OsDHAR* gene in the *S. elongatus* PCC 7942. **(A)** Schematic showing expression of *OsDHAR* in of *S. elongatus* PCC 7942. *Ptrc*, IPTG-inducible promoter; *OsDHAR*, dehydroascorbate reductase gene of *O. sativa*; NAT, antibiotic nourseothricin selection marker (cloNAT); and *LacIq*, lactose repressor promoter. Arrows indicate the direction of each gene. **(B,C)** PCR results verifying *OsDHAR* gene integration in the genome of the transgenic TA. **(B)** PCR of *OsDHAR* gene in transgenic TA to confirm genotype (690 bp). **(C)** Expression of the *OsDHAR* gene was confirmed by semi-quantitative RT-PCR (247 bp) and Western blot (23.5 kDa). N, normal conditions; S, stress conditions. The *rpoA* transcript was used as a control standard in the analysis (250 bp). **(D,E)** Growth profiles of the TA, EV, and WT *S. elongatus* PCC 7942. Growth levels of the different *S. elongatus* PCC 7942 were compared to determine whether the TA grew better under H_2_O_2_ stress conditions. After 7 days of growth under standard conditions, the TA, EV, and WT *S. elongatus* PCC 7942 were adjusted to an absorbance at 730 nm of 0.3 to 0.35 and then grown in BG11 medium containing 2.5 mM H_2_O_2_, then cultured for 18 days at 30°C with shaking in liquid microbial lab flasks. WT, WT *S. elongatus* PCC 7942; EV, empty vector *S. elongatus* PCC 7942; TA, TA *S. elongatus* PCC 7942. Error bars indicate ± SD of biologically least three independent experiments. Asterisks indicate significant differences between treatments as estimated by Student’s *t*-test (*P* < 0.05).

The expression of heterologous *OsDHAR* in *S. elongatus* PCC 7942 was constructed to evaluate the effect on tolerance to hydrogen peroxide (H_2_O_2_)-induced oxidative stress. *OsDHAR* was cloned into the pAM4957 (pCV0069) vector for NS2 chromosomal integration under the control of the IPTG-inducible promoter (*Ptrc*) (Figure 2A). Resistance to oxidative stress was evaluated in cyanobacterial *S. elongatus* PCC 7942 conjugated with the *pAM4957*:*OsDHAR* plasmid containing the *OsDHAR* gene or the pAM4957 vector alone plasmid containing the empty neutral site vector (EV) (Supplementary Table S1). Genotypes were confirmed by PCR using primers external to the *OsDHAR* gene (Supplementary Table S2) that produced the expected fragment size of 690 bp (Figure 2B and Supplementary Table S1).

We explored whether the *OsDHAR* gene was effectively expressed with a semi-quantitative RT-PCR analysis using primers internal to the *OsDHAR* gene (Supplementary Table S2). The *OsDHAR* gene was expressed in the TA, while no signal was detected in the empty vector (EV, pAM4957) or the wild-type (WT) *S. elongatus* PCC 7942 (Figure 2C and Supplementary Table S1). We also performed Western blot analyses to determine whether the *OsDHAR* gene was properly translated. The TA grown under H_2_O_2_ stress conditions presented with a single band in a Western blot analysis using the OsDHAR antibody. No signal was detected in the EV and WT under the same conditions (Figure 2C). These results indicated that the *OsDHAR* gene under the control of the *Ptrc* promoter was expressed in the TA during incubation in 2 mM IPTG at 30°C for 2 days before 2.5 mM H_2_O_2_ treatment.

Stress tolerance was measured based on growth kinetics, colony formation on plates, and chlorophyll content. The TA grew better than the EV and WT under H_2_O_2_ stress conditions (Figure 2E). The WT and control EV showed loss of pigment on days 7, 8, and 10 after exposure to H_2_O_2_ stress, and were completely bleached by day 12, whereas the TA showed loss of pigment on day 10 (Figure 2D).

**FIGURE 3:**
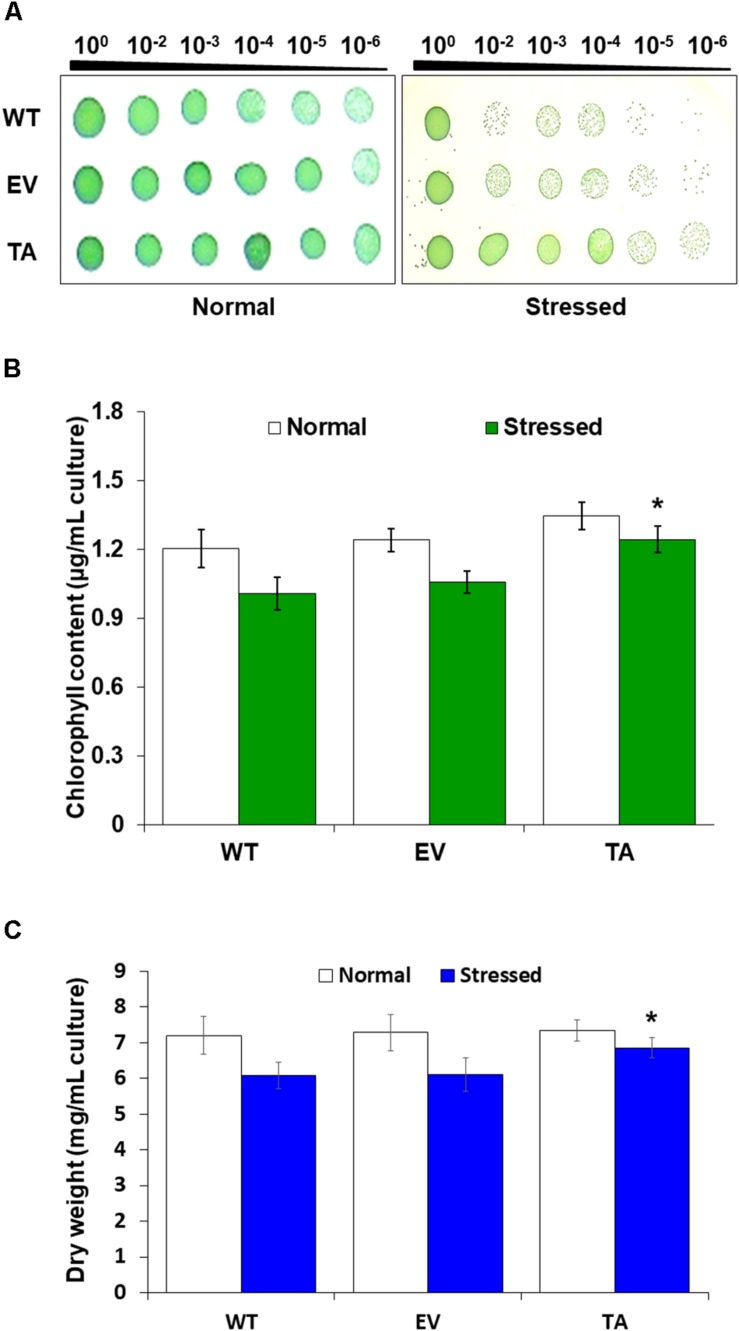
Stress sensitivity in the *OsDHAR*-expressing transgenic TA. **(A)** Cell viability was evaluated based on number of colony-forming units and spotting assay. **(B)** Chlorophyll contents of grown for 2 days in the presence of 2.5 mM H_2_O_2_ (*A*_730_ = 0.3). **(C)** Dry weights measured at 7 and 9 days after growth in the presence or absence of 2.5 mM H_2_O_2_ for 2 days. Error bars indicate ± SD of biologically least three independent experiments. Asterisks indicate significant differences between treatments as estimated by Student’s *t*-test (*P* < 0.05). WT, WT *S. elongatus* PCC 7942; EV, empty vector *S. elongatus* PCC 7942; TA, TA *S. elongatus* PCC 7942.

Stress responses were also evaluated with a plate cell-spotting assay. Exponentially growing TA, EV, and WT (absorbance at 730 nm = 0.25) were exposed to 2.5 mM H_2_O_2_ with 2 mM IPTG for 3 days, and then serial dilutions were spotted on agar-solidified BG11 medium. Cell viability of the TA was better than that of the EV and WT following exposure to H_2_O_2_ stress. On the other hand, all individuals were similarly spotted under normal conditions (Figure 3A). Measurement of chlorophyll content after 2 days exposure to H_2_O_2_ revealed less loss of chlorophyll in the TA than in the EV and WT (Figure 3B). The total dry weight of the TA was twofold higher than that of the EA and WT under oxidative stress conditions (Figure 3C).

**FIGURE 4:**
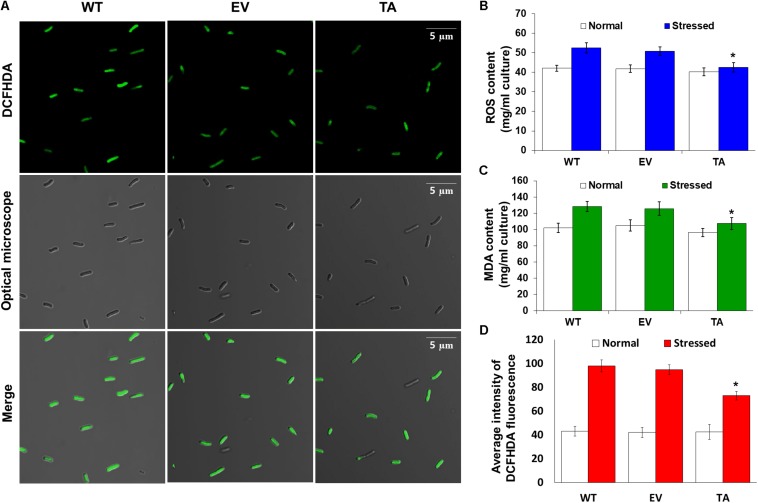
Expression of the *OsDHAR* gene in *S. elongatus* PCC 7942 improves intracellular redox homeostasis. **(A)** Confocal microscopy images of cells stained with DCFHDA to measure levels of ROS after treatment with 2.5 mM H_2_O_2_ for 2 h. **(B)** Intracellular ROS levels were determined using intracellular H_2_O_2_ after treatment with 2.5 mM H_2_O_2_ for 24 h. **(C)** Levels of lipid peroxidation were determined by TBA assay using MDA after treatment to 2.5 mM H_2_O_2_-IPTG with 2 mM IPTG for 24 h. **(D)** DCFHDA fluorescence they also measured ROS production via a microplate reader after treatment with 2.5 mM H_2_O_2_ for 2 h. Error bars indicate ± SD of biologically least three independent experiments. Asterisks indicate significant differences between treatments as estimated by Student’s *t*-test (*P* < 0.05).

Redox states were determined based on cellular ROS levels assayed with the cytosolic oxidant-sensitive probe 2′,7′-dichlorodihydrofluorescein diacetate (DCFHDA), which measures the oxidative conversion of DCFHDA to the highly fluorescent compound dichlorofluorescein (DCF). All three the TA, EV, and WT *S. elongatus* PCC 7942 showed an increase in DCF fluorescence following exposure to 2.5 mM H_2_O_2_ with 2 mM IPTG for 2 h, but the fluorescence intensity of the probe was more pronounced in the control EV and WT than in the TA (Figure 4A). When the TA, EV, and WT were subjected to oxidative stress, the TA displayed ROS-mediated cellular damage with or without ROS generation and contained moderate green fluorescence in the intracellular space. In contrast, EV and WT showed much more intense green fluorescence (Figure 4A). DCFHDA fluorescence was quantified by individually mixing the TA, EV, and WT with 10 μM DCFHDA followed by incubation for 20 min in the dark. Fluorescence intensity was 1.5-fold higher in the EV and WT than in the TA in the presence of 2.5 mM H_2_O_2_ with 2 mM IPTG (Figure 4D). In a biochemical assay using the FOX reagent, cellular ROS levels in the TA were determined to be approximately 20% lower than those in the EV and WT in the presence of H_2_O_2_ (Figure 4B). The lower ROS production in the TA resulted in reduced accumulation of MDA due to lipid peroxidation compared to the EV and WT. MDA accumulations were 1.2-fold higher in the EV and WT than in the TA (Figure 4C).

### Effect of *OsDHAR* Expression on the GSH-Dependent Antioxidant System

**FIGURE 5:**
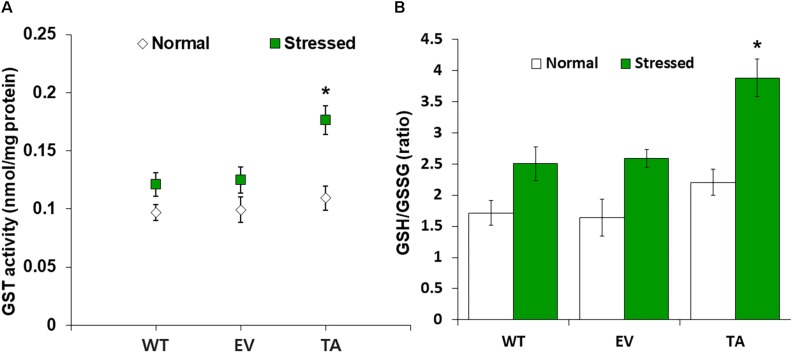
Analysis of enzyme activity and antioxidant redox status in TA after H_2_O_2_ treatment. **(A)** Activity of GST under normal and stress conditions. **(B)** Intracellular GSH/GSSG redox ratios in WT, EV, and TA after exposure to 2.5 mM H_2_O_2_ for 24 h. White bars correspond to conditions before H_2_O_2_ treatment (normal conditions), and the GSH/GSSG redox ratios (green bars) correspond to conditions after H_2_O_2_ treatment (stress conditions). Data are shown as mean values ± SD of biologically least three independent experiments. Asterisks indicate significant differences between treatments as estimated by Student’s *t*-test (*P* < 0.05). Measurements are an annotated in Supplementary Figure S1.

We analyzed redox homeostasis in the *OsDHAR*-expressing transgenic by measuring the GSH pools to determine how *OsDHAR* expression affects total GSH content in cyanobacteria exposed to H_2_O_2_. In the TA, GSH redox status was expressed as the ratio of reduced GSH to oxidized GSSG (GSH/GSSG), was approximately 1.5-fold higher than those of the EV and WT (Figure 5B). The GSH/GSSG ratio in the TA were higher under oxidative stress than under normal conditions. Similarly, the GSH/GSSG ratio in the EV and WT were also moderately increased under oxidative stress, although overall values were lower than those of the TA (Figure 5B). Conversely, oxidized GSSG content in the EV and WT increased tremendously over baseline values compared to the TA under oxidative stress and normal conditions (Supplementary Figure S1).

**FIGURE 6:**
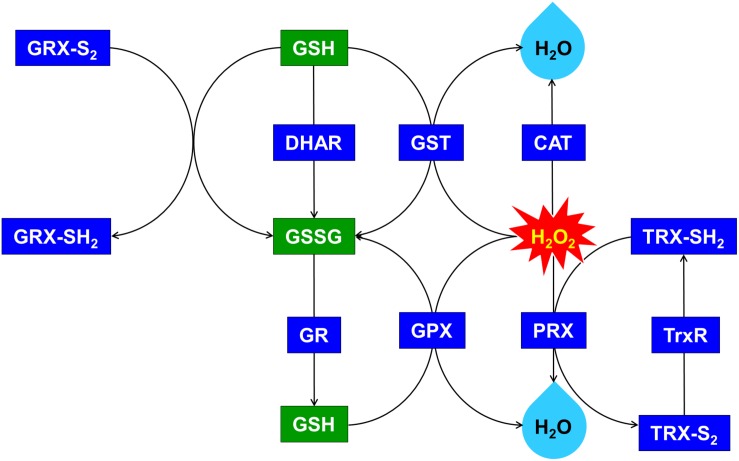
Upregulated glutathione (GSH)-dependent antioxidant system and maintenance of cellular redox status through the overexpression of *OsDHAR* in *S. elongatus*. GRX, glutaredoxin; GST, glutathione *S*-transferase; GR, glutathione reductase; GPX, glutathione peroxidase; CAT, catalase; PRX, peroxiredoxin; TRX, thioredoxin; TrxR, thioredoxin reductase. qRT-PCR identified GSH-related genes that were annotated in Supplementary Figure S3.

*OsDHAR* expression in the TA was also associated with increased GST activity under oxidative stress conditions (Figure 5A). These results indicate that enhanced resistance to the oxidative stress by maintaining of the cellular redox homeostasis during ROS-induced oxidative stress relates to the homology GST in cyanobacteria. For this reason, increased homeostasis correlated with total GSH content, and oxidized GSH and reduced GSH were observed in cyanobacteria harboring the *DHAR* gene. This result strongly suggests that the expression of DHAR (with its homology with GST) in *S. elongatus* PCC 7942 enhances tolerance of ROS-induced oxidative stress by maintaining cellular redox homeostasis using GSH alongside other antioxidant enzymes in cyanobacteria (Figure 6 and Supplementary Figure S3). Thus, heterologous *OsDHAR* expression in the TA responded to oxidative stress by increasing the number of redox active sites, such as GSH binding sites, and GSTs with similar functionally conserved residues under these conditions (Figures 1, 5). Altogether, our findings indicate that *OsDHAR* expression conferred tolerance to oxidative stress in *S. elongatus* PCC 7942 by interacting with GSH-dependent enzymes.

We explored this possibility further by conducting gene expression of the TA after exposure to H_2_O_2_-IPTG for 24 h. qRT-PCR identified GSH-dependent genes that were expressed in the TA after treatment with 2.5 mM H_2_O_2_-IPTG for 24 h in liquid BG11 medium containing 2 mM IPTG (Supplementary Figure S3). These genes included GST, thioredoxin reductase (TrxR), glutathione reductase (GR), catalase (CAT), glutathione peroxidase (GPX), 2-Cys peroxiredoxin (TRX), glutaredoxin (GRX), and peroxiredoxin (PRX) belonging to GSH-dependent reactions (Figure 6 and Supplementary Figure S3). Our results indicate that heterologous *OsDHAR* expression in the TA can enhance tolerance through the maintenance of cellular redox homeostasis via the GSH-dependent antioxidant system during ROS-induced oxidative stress.

## Discussion

Heterologous OsDHAR and the other GSTs demonstrated pairwise alignment with a conserved site that bound to GSH residues and other similarly functional conserved residues (Figure 1). OsDHAR also contains interacting residues of the GSH binding site. This indicates that OsDHAR can supply a redox active site during ROS-induced oxidative stress (Pulido et al., 2010; Do et al., 2016; Kim et al., 2017). The similarity of the aforementioned conserved DHAR sequences indicated that the OsDHAR redox active site possesses strictly conserved residues, including a GSH binding site and similar functional conserved residues from cyanobacteria GSTs. This GSH-dependent antioxidant system can facilitate GSH-dependent reactions during ROS-induced oxidative stress (Figure 6 and Supplementary Figure S3). As such, the redox active sites of OsDHAR participate in the maintenance of a stable cellular thiol/disulfide ratio, redox regulation, and refolding of oxidatively damaged proteins (Thomas et al., 1999; Hamnell-Pamment et al., 2005; Latifi et al., 2009; Anfelt et al., 2013; Kim et al., 2013; Abdul Kayum et al., 2018). In addition, we constructed a phylogenetic tree containing the *S. elongatus* PCC 7942 GST (SeGST; accession no. WP_011243077), which was 24% homologous to OsDHAR (accession no. AAL71856.1) (Figure 1B). The phylogenetic tree illustrated the evolutionary relationship between rice DHAR and cyanobacterial GST proteins.

We analyzed *OsDHAR*-mediated stress tolerance in transgenic *S. elongatus* PCC 7942 under H_2_O_2_ stress conditions because excess H_2_O_2_ is a major cause of oxidative stress in biological systems, and cyanobacteria are generally more sensitive to H_2_O_2_ than are other phototrophs (Drabkova et al., 2007; Banerjee et al., 2015). We investigated the mechanism by which *OsDHAR* gene overexpression increased H_2_O_2_ stress tolerance by examining the phenotypes and gene expression of the TA under H_2_O_2_ stress. There were no differences in phenotype among the TA, EV, and WT *S. elongatus* PCC 7942 grown under normal conditions (7 days; Figure 2D). Under H_2_O_2_ stress, however, most of the EV and WT had lost almost all their pigment after 10 days, while the TA lost considerably less pigment over this same period. The TA also recovered quickly from H_2_O_2_ damage (12 days; Figures 2D,E) and overall TA survival rates were higher than those of the EV and WT. Results also demonstrated that the TA, EV, and WT possessed clear phenotypic differences that correlated with their ability (or inability) to recover from H_2_O_2_ damage. Specifically, the TA recuperated quickly after H_2_O_2_ treatment, whereas the EV and WT did not (Figures 2D,E).

Stress responses were also evaluated by plate cell-spotting assay following H_2_O_2_ stress. Cell viability of the TA was better than the EV and WT following exposure to 2.5 mM H_2_O_2_ for 3 days (Figure 3A). Measurement of chlorophyll content after a 2-day exposure to 2.5 mM H_2_O_2_ revealed a more loss of 18% less chlorophyll in the EV and WT compared to the TA (Figure 3B). Based on these results, the total dry weight of the TA was twofold higher than the EA and WT to H_2_O_2_ stress (Figure 3C).

**FIGURE 7:**
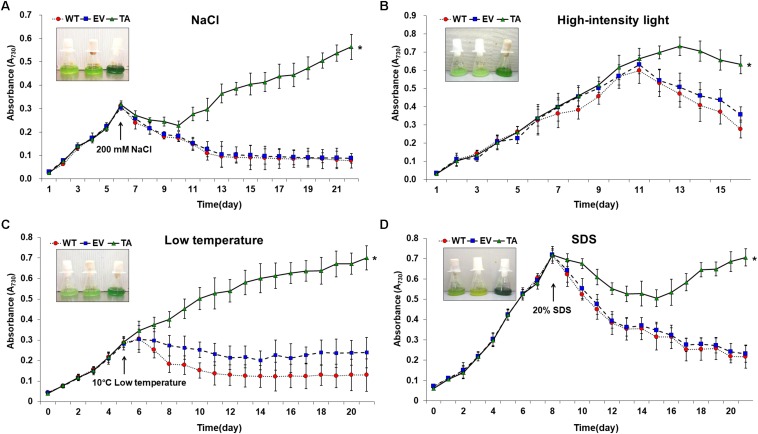
Growth profiles of WT, EV, and TA in the presence of oxidative stressors, including **(A)** 200 mM NaCl after incubation for 6 days, **(B)** 250 μE high-intensity light during continuous incubation, **(C)** low temperature (10°C) after incubation for 5 days, and **(D)** 20% SDS after incubation for 8 days. Photographs were taken approximately 3 days after stress treatments at which point the sensitive phenotype appeared. Error bars indicate ± SD of biologically least three independent experiments. Asterisks indicate significant differences between treatments as estimated by Student’s *t*-test (*P* < 0.05). WT, WT *S. elongatus* PCC 7942; EV, empty vector *S. elongatus* PCC 7942; TA, TA *S. elongatus* PCC 7942.

The TA, EV, and WT were additionally exposed to oxidative stress due to high salt concentrations (200 mM NaCl), low temperature (10°C), excess light (250 μE high-intensity light), and sodium dodecyl sulfate (SDS, 20%) (Figure 7). When cells are exposed to high salinities or low temperatures, major processes of photosynthesis, protein synthesis, and energy and lipid metabolism are slowed or halted (Demiral and Turkan, 2006). In addition, the stress of excess light can induce damage to the reaction center of photosystem II and may lead to the perturbation or inhibition of photosynthetic electron transport in cells (Fryer et al., 2003; Kim et al., 2018). Previous studies have shown that multiple ion channel pores in cell participate to stress responses in cyanobacterial cells (Los and Murata, 2004; Kim et al., 2018). SDS and cold stress were a surfactant that damage lipid membranes, thereby disrupting cellular redox homeostasis and leading to apoptosis (Kwon et al., 2003; Tahara et al., 2012; Shin et al., 2013).

The TA displayed better growth than the control EV and WT in the presence of NaCl, high-intensity light, low temperature, and SDS (Figure 7). These results indicate that the expression of the *OsDHAR* gene in the TA conferred higher tolerance to oxidative stresses compared to that of the EV and WT. As already mentioned, DHAR participates in cellular detoxification, stress tolerance, and light signaling (Loyall et al., 2000; Thom et al., 2002; Benekos et al., 2010). Thus, DHAR as isozymes are critical to cellular detoxification of exogenous and endogenous harmful compounds. In aerobic cells, high DHAR activity enhances growth and development through increased chlorophyll fluorescence under oxidative stress, whereas it decreases chlorophyll in aging cells under the same conditions (Wang et al., 2010; Kim et al., 2013; Do et al., 2016). In cyanobacteria, H_2_O_2_-induced oxidative stress and the resulting excess ROS lead to cell death in several types of cyanobacteria (Drabkova et al., 2007; Qian et al., 2010; Banerjee et al., 2015). We investigated the effects of oxidative stress produced by H_2_O_2_-generated ROS by analyzing ROS levels *in vitro* and *in vivo* in the TA, EV, and WT. Cellular ROS levels were assayed to evaluate cell rescue redox states with the cytosolic oxidant-sensitive probe 2′,7′-dichlorodihydrofluorescein diacetate (DCFHDA), which measures the oxidative conversion of DCFHDA to the highly fluorescent compound dichlorofluorescein (DCF). The TA displayed ROS-mediated cellular damage with and without the targeting signal, and contained weaker green fluorescence in the intracellular space than the EV and WT in the presence of 2.5 mM H_2_O_2_ (Figure 4A). DCFHDA fluorescence intensity was 1.5-fold higher in the EV and WT than the TA in the presence of 2.5 mM H_2_O_2_ (Figure 4D). Cellular ROS levels in the TA were found to be lower than those in the EV and WT through a biochemical assay using the FOX reagent in the presence of H_2_O_2_ (Figure 4B). Lower ROS production in the TA was associated with reduced accumulation of MDA due to lipid peroxidation compared to the EV and WT (Figure 4C). These results indicated that transgenic *OsDHAR* expression in the TA increased cell tolerance to oxidative stress by scavenging ROS and increasing chlorophyll content. These findings indicate that *OsDHAR* is key to oxidative tolerance through the maintenance of cellular redox homeostasis via a GSH-dependent antioxidant system.

Cells employ several mechanisms to combat toxic ROS generated by abiotic stresses like oxidants. The generated ROS can be scavenged by non-enzymatic antioxidants, such as GSH, or by enzymatic systems, such as the GSH pools involving DHAR (Noctor and Foyer, 1998; Kim et al., 2014). Different environmental stressors, including high salinity, low temperature, and drought, can activate *DHAR* expression, which goes on to regulate GSH pools (Urano et al., 2000; Jiang and Zhang, 2002; Kwon et al., 2003; Eltayeb et al., 2007; Gill and Tuteja, 2010). GSTs have been divided into eight classes: tau, phi, theta, zeta, lambda, EF1Bγ, DHAR, and tetrachlorohydroquinone dehalogenase (TCHQD) (Oakley, 2005; Lan et al., 2009; Dixon and Edwards, 2010; Jain et al., 2010; Liu et al., 2013).

Among these, DHAR and GST share considerable homology has been ubiquitously produced in eukaryotes and prokaryotes, such as plants, animals, fungi, and bacteria (Dixon et al., 2002; Liu et al., 2013; Rezaei et al., 2013; Ding et al., 2017). The homologous DHAR and GST have similar functions involving their redox active sites and GSH binding site residues (Dixon et al., 2002; Kim et al., 2013). Both DHAR and GST execute GSH-dependent thiol transferase activity to recycle antioxidants, including flavonoids, and quinones (Dixon et al., 2011). In this study, GST activity increased concomitantly with GSH via heterologous *OsDHAR* overexpression in cyanobacteria (Figure 5 and Supplementary Figure S1). Cellular oxidative stress depends on maintaining a balance between ROS content and the antioxidant system (Tang et al., 2007; Kim et al., 2017, 2018). Increased production of antioxidants (GSH) and antioxidant enzymes (GST) can ameliorate excess ROS during oxidative stress. In this study, these phenomena were associated with TA resistance to oxidative stress (Figures 2–7 and Supplementary Figure S1). We thereby conclude that the expression of heterologous *OsDHAR* in *S. elongatus* PCC 7942 enhanced tolerance to ROS-induced oxidative stress by maintaining cellular redox homeostasis through cooperation of the homologous GST in cyanobacterial cells.

Previous evidences have established a relationship between stress tolerance and the cellular redox homeostasis that is affected by *DHAR* overexpression in several cells (Horemans et al., 2000; Kwon et al., 2003; Ushimaru et al., 2006; Eltayeb et al., 2007; Qian et al., 2010; Wang et al., 2010). We confirmed that the TA was resistant to H_2_O_2_ stress and that this was related to an increased GSH/GSSG ratio (Figure 5B) and GST activity (Figure 5A). Our results indicated that heterologous *OsDHAR* expression in *S. elongatus* PCC 7942 confers tolerance to oxidative stress by interacting with GSH-dependent enzymes. Gene expression levels of the TA during exposure to H_2_O_2_ was also conducted to confirm this conclusion, and qRT-PCR identified GSH-related genes that were upregulated, including GST, TrxR, GR, CAT, GPX, TRX, GRX, and PRX, belonging to GSH-dependent reactions in the TA (Figure 6 and Supplementary Figure S3). GSH-dependent antioxidation involving GST, GR, CAT, GPX, GRX, TrxR, PRX, and TRX are all involved in protecting cells from oxidative stress and minimizing subsequent cellular damage (Kingston-Smith and Foyer, 2000). In particular, the GSH-dependent enzymes are among the most potent protein-based protection mechanisms and occur in nearly all cells, from bacteria like *E. coli* to higher plants and animals (Meyer et al., 2012). We surmise that oxidative stress tolerance in the TA would be increased through improved maintenance of redox homeostasis compared to the WT and EV (Figures 2–7 and Supplementary Figure S3).

Taken together, our findings indicate that the expression of *OsDHAR* in *S. elongatus* PCC 7942 maintained cellular redox homeostasis and protected cells from oxidative damage with the GSH-dependent antioxidant system via GSH-dependent reactions at the redox active site and GSH binding-site residues. However, genetic and molecular underpinnings of heterologous *OsDHAR* and homologous *SeGST* activity remain unclear. Cellular redox homeostasis and molecular processes involved in their response to various environmental conditions warrant further study.

We found that expression of the *OsDHAR* gene in the *S. elongatus* PCC 7942 increased tolerance to ROS-induced oxidative stress through the maintenance of cellular redox homeostasis with a GSH-dependent antioxidant system via GSH-dependent reactions. We anticipate that other antioxidant defense mechanisms might act synergistically to produce an effective defense response and protect cells during oxidative stress. The genetically modified cyanobacteria used in this study could help verify mutual functions between *O. sativa DHAR* and *S. elongatus* PCC 7942 *GST* genes. Further studies are needed to elucidate the mechanisms that underlie the increased oxidative stress tolerance observed in heterologous *OsDHAR*-expressing transgenic cyanobacterium.

## Data Availability Statement

The raw data supporting the conclusions of this article will be made available by the authors, without undue reservation, to any qualified researcher.

## Author Contributions

Y-SK, I-SK, AT, JG, and H-SY designed the experiments. S-IP, J-JK, and K-IL performed the experiments. The article was written by Y-SK, and edited by JSB and JB. All authors read and approved the final manuscript.

## Conflict of Interest

The authors declare that the research was conducted in the absence of any commercial or financial relationships that could be construed as a potential conflict of interest.

## Footnotes

1 http://clustalw.ddbj.nig.ac.jp/
