# Supplementary material for: Expression of Heterologous OsDHAR Gene Improves Glutathione (GSH)-Dependent Antioxidant System and Maintenance of Cellular Redox Status in Synechococcus elongatus PCC 7942
Source: Front Plant Sci. 2020 Mar 3;11:231. doi: 10.3389/fpls.2020.00231 (PMC7063034; doi:10.3389/fpls.2020.00231)
Supplement: Supplementary file 1 [file Data_Sheet_1.PDF]

Supplementary Materials for

Expression of Heterologous *OsDHAR* Gene Improves Glutathione (GSH)-Dependent Antioxidant System and Maintenance of Cellular Redox Status in *Synechococcus elongatus* PCC 7942

Young-Saeng Kim[1], Seong-Im Park[3,4], Jin-Ju Kim[3,4], Joseph S. Boyd[6], Joris Beld[7], Arnaud Taton[6], Kyoung-In Lee[5], Il-Sup Kim[2,*], James W. Golden[6,*], Ho-Sung Yoon[2,3,4 *]

[1]Research Institute for Dok-do and Ulleung-do, Kyungpook National University, Daegu 41566, Republic of Korea. [2]Advanced Bio-resource Research Center. [3]Department of Biology, Kyungpook National University, Daegu 41566, Republic of Korea. [4]School of Life Sciences, BK21 Plus KNU Creative BioResearch Group, Kyungpook National University, Daegu 41566, Republic of Korea. [5]Biotechnology Industrialization Center, Dongshin University, Naju 58205, Republic of Korea. [6]Division of Biological Sciences, University of California San Diego, La Jolla, CA 92093-0116, USA. [7]Department of Microbiology and Immunology, College of Medicine, Drexel University, PA 19102, USA.

Tel.: +82-53-951-5348
* Correspondence: Il-Sup Kim: 92kis@hanmail.net; James W. Golden: jwgolden@ucsd.edu; Ho-Sung Yoon: hsy@knu.ac.kr

Supplementary Figures S1 and S3

Supplementary Tables S1 to S2

Supplementary Figure S1.

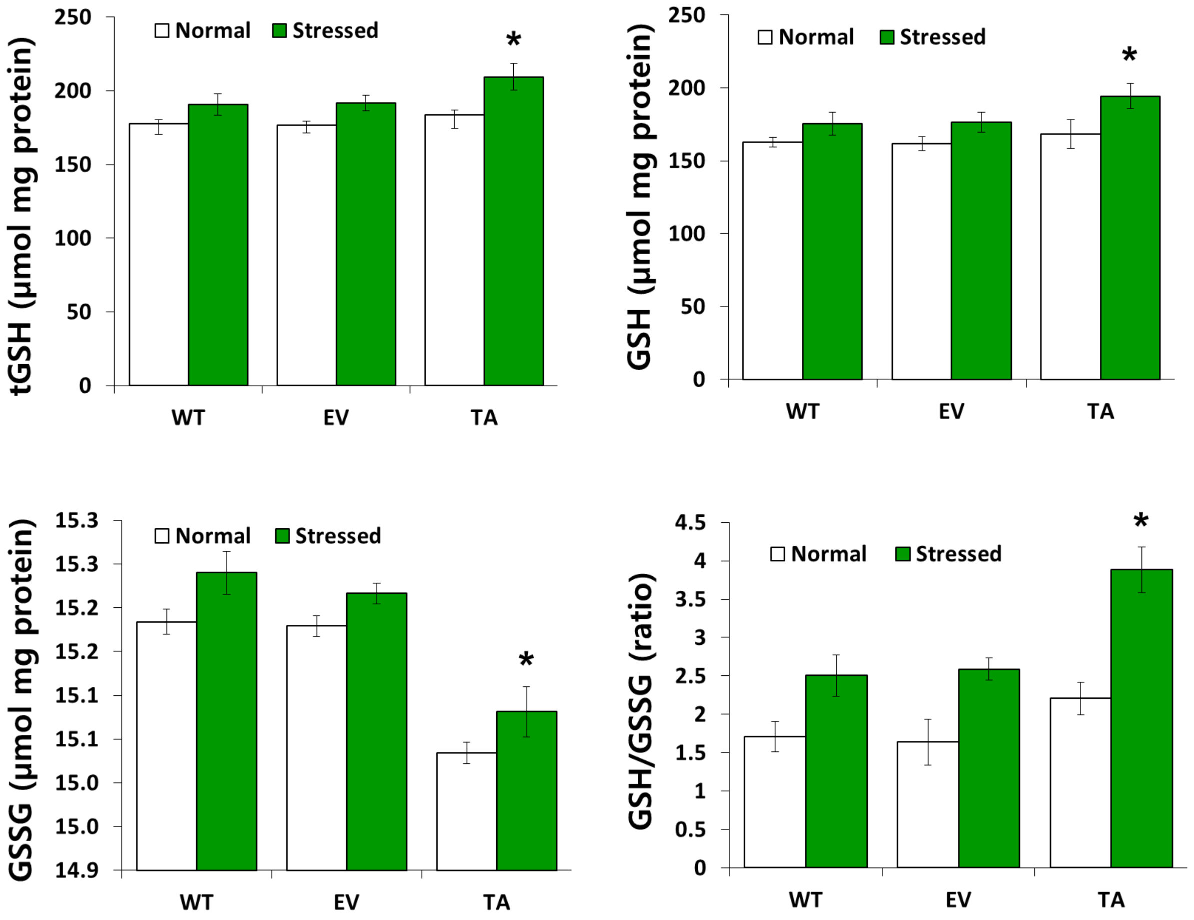

Supplementary Figure S1. Analysis of antioxidant redox status in TA strains after H_2O_2 treatment. Total glutathione (GSH+GSSG) and oxidized glutathione (GSSG) were determined by measuring absorbances at 412 nm. Reduced GSH content was calculated by subtraction of GSSG from total GSH. Total glutathione (GSH+GSSG), reduced glutathione (GSH), oxidized glutathione (GSSG), and ratio of GSH to GSSG. Error bars indicate ± SD of three independent experiments. Asterisks indicate significant differences between treatments as estimated by Student's *t*-test ($P < 0.05$).

Supplementary Figure S2.

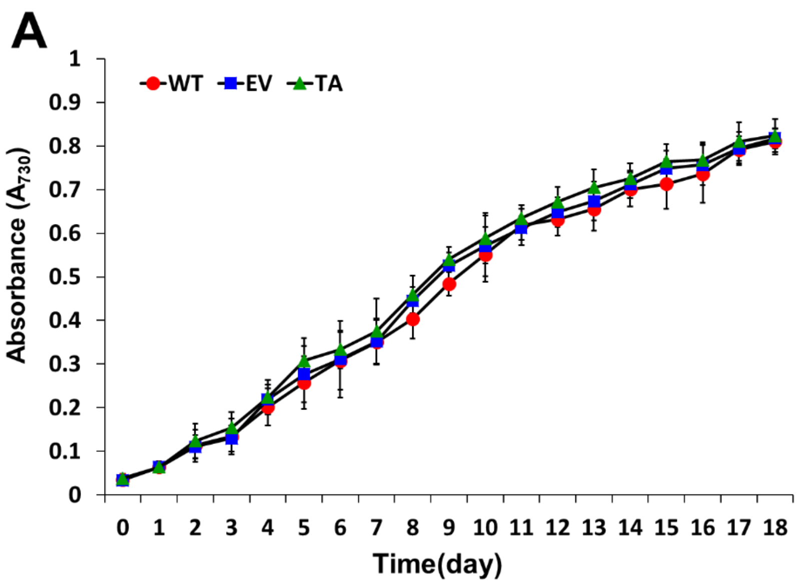

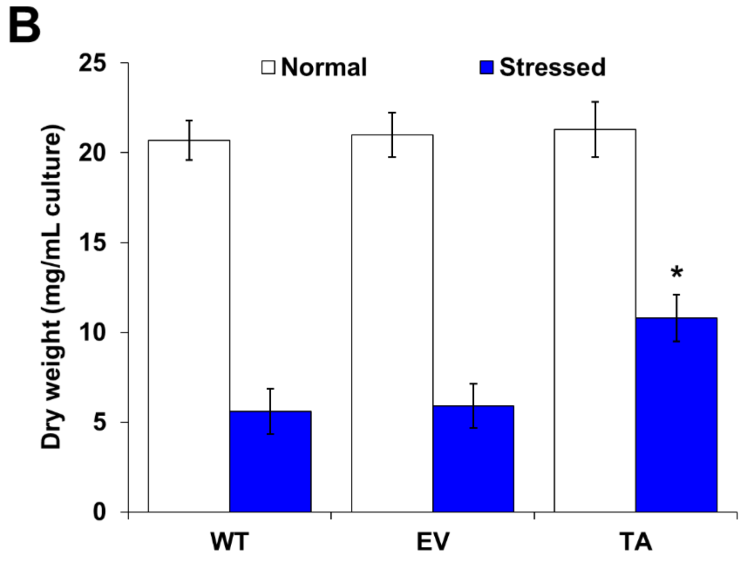

Supplementary Figure S2. Dry weights of *OsDHAR*-overexpressing transgenic TA strains under normal and stress conditions. **(A)** Growth profiles of WT, EV, and TA strains. growth levels of the different *S. elongatus* PCC 7942 strains were compared to those of the TA strain under normal conditions. Samples were grown for 18 days at 30°C with shaking under normal conditions. **(B)** Dry weights measured after growth under normal condition for 14 days and stress conditions for 7 days after H_2O_2 treatment at 7 days. WT, WT strains; EV, empty vector strains; TA, transgenic strains. Error bars indicate ± SD of three or more independent experiments. Asterisks indicate significant differences between treatments as estimated by Student’s *t*-test ($P < 0.05$).

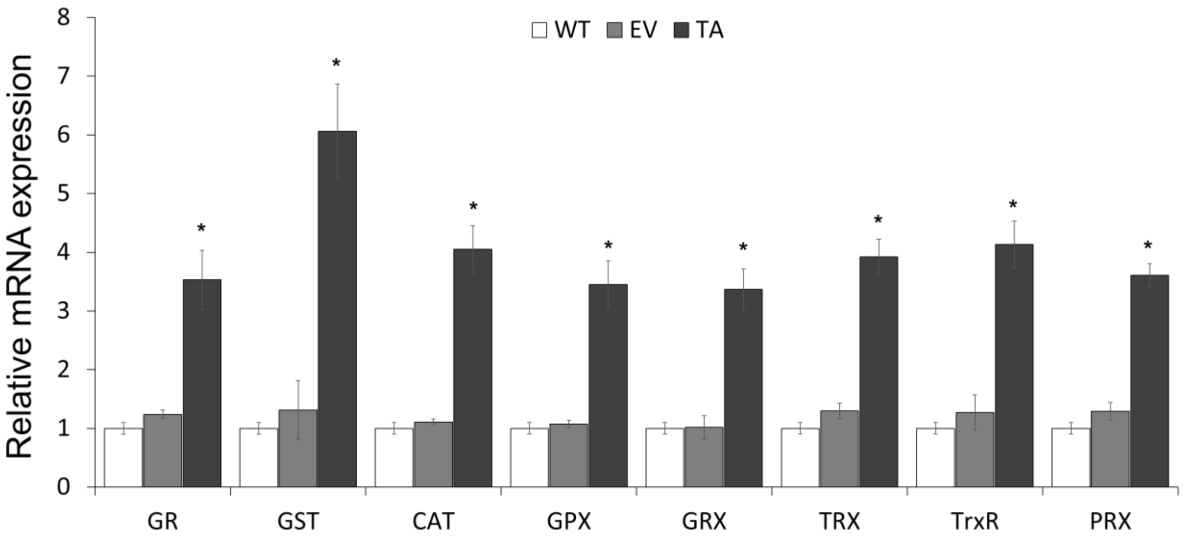

Supplementary Figure S3. Expression analysis of qRT-PCR identified GSH-related genes. GSH-related genes expression level was considered to be 1 of WT strains; described as relative values. GSH-related genes expression difference was calculated relative to the expression level in WT strains. WT, WT strains; EV, empty vector strains; TA, transgenic strains. Error bars indicate ± SD of three or more independent experiments. Asterisks indicate significant differences between treatments as estimated by Student's *t*-test ($P < 0.05$).

Supplementary Table S1. Cyanobacterial strains and plasmids used in this study.

Strain or Plasmid	Characteristic(s)	Antibiotic resistance	Source or reference
**Strains**			
AMC06	Wild type *Synechococcus elongatus* strain PCC 7942	none	Lab collection
DH5α	*Escherichia coli* cloning host	none	Lab collection
**Plasmids**	Relevant characteristics*		
pAM4957 (pCV0069)	*S. elongatus* carrying *pCV0069* as a single recombinant; cloNAT^r	NAT	Lab collection
pAM4957::OsDHAR	S7942NS2-NAT1-Ptrc/SwaI-OsDHAR-RBS_*OsDHAR*, 5672 bp, cloNAT^r , for IPTG-inducible *OsDHAR* expression	NAT	This work

* Abbreviations for antibiotics: Nourseothricin (NAT, cloNAT).

Supplementary Table S2. Primers used in this study.

Name	Sequence
OsDHAR-F-SwaI	5´-TGCGGATCCTAAGGAGGAAAATTTATGGGCGTGGAGGTGTGCGTCAAGGCCGCCGTCG-3´
OsDHAR-R-SwaI	5´- GCCGGGGAGCTCCTTCATTTATTTTTACGCATTCACTTTTGGTGCCCATCCAGCAATCAGG-3´
OsDHAR-qRT-PCR-F	5’-ATGGGCGTGGAGGTGTGCGTCAAGGCCGCC-3’
OsDHAR-qRT-PCR-R	5´-TGACAAGAGACGGGGTTGGGTAC-3´
rpoA-F	5´-TCGGTTCGGGCTTACAACTG-3´
rpoA-R	5´-AGCAAGTCAGCAACGGAGTTC-3´
NS2-F	5’-GTCAACGTAAATGCATGCCGC-3’
NS2-R	5’-GGTCACTACTTGGTAGCAACTC-3’
Ptrc-F	5´-AATTGTGAGCGGATAACAATT-3´
OsDHAR-R	5´-ATTTATTTTTACGCATTCACTTTTGG-3´
GR-F	5´-ATGAGTTTTGACTACGACCTC-3´
GR-R	5´-GCCCCCGACCAAATCGGTTTC-3´
GST-F	5´-ATGTATAAGGTTTTCGGAGA-3´
GST-R	5´-ATGAGTTTCGTTAGCAAGTA-3´
CAT-F	5´-ATGACAGTCGTTATCTACTAC-3´
CAT-R	5´-GATCGCTAACCAACGAAACCA-3´
GPX-F	5´-ATGCCTTTCAAAGCAAAAGA-3´
GPX-R	5´-TGTTTGACAGAAAGGACAAA-3´
GRX-F	5´-ATGATGACTCCTGAACTCC-3´
GRX-R	5´-TTGAACAACGTTGTTGGAA-3´
TRX-F	5´-ATGAACGTTGGCGATCGCGTGC-3´
TRX-R	5´-AGTTAGAATCGCTGCAATTTCG-3´
TrxR-F	5´-ATGCTTCCAGTCGTCACAGC-3´
TrxR-R	5´-GAGGCAGGGAGTTAAGAGTC-3´
PRX-F	5´-ATGACCGAAGGAGCCCTGCGCG-3´
PRX-R	5´-CAGAACGACGTATTTGCCCCGG-3´
